# Resection of the Optic Nerve

**Published:** 1891-07

**Authors:** 


RESECTION OF THE OPTIC NERVE. By L. Webster Fox, M. D. Philadelphia: Reprinted from the *Medical and Surgical Reporter*, February 7th, 1891.

This little pamphlet is a protest against the practice of enucleation of an injured eyeball to prevent sympathetic ophthalmia. Instead of cutting out the useless eye and replacing it with a glass imitation, he proposes to retain it and simply resect the optic nerve.

The details of the operation for resection are essentially as follows: The lids are separated with the ordinary ophthalmostat; a vertical incision is made through the conjunctiva over the insertion of the external rectus muscle; the conjunctiva is dissected off as far back as the external canthus will permit. This exposes completely the muscle. Two silk threads are then passed through the muscle near its tendinous insertion. These threads will be required in a subsequent stage of the operation, to unite the detached muscle to the eyeball. The muscle is then cut and drawn to the temple side. This exposes the globe. With curved scissors all tissue is then separated from the eyeball. The optic nerve is found by passing a hook—which is nothing more than a strabismus hook bent at a right angle. This hook brings forward the nerve, and then the retractor is passed downwards until it meets the nerve, and is then passed down and out, keeping the adipose tissue out of the way. A second bent hook is inserted under the optic nerve, and also pressed backward. A certain portion of the nerve becomes exposed, and with a delicate flat forceps the nerve is grasped and held firmly. This is to prevent hemorrhage from the ophthalmic vein and artery (central) after the cut with the scissors. The eyeball is rotated forward, so that the non-severed nerve becomes exposed and a small piece of its bulbar end is cut off. By keeping pressure on the orbital end of the nerve for a few minutes, all danger of hemorrhage is aborted. The eyeball is then rotated into place, and the external muscle is re-adjusted. Over this the conjunctiva is replaced, and it is held in position with black silk, which may be removed in three days. Antiphlogistic dressings are applied day and night for several days. Very little reaction follows and in a week or ten days the eye has assumed its normal appearance with no disfigurement, and the action of the muscle is complete.

